# A novel immune-related microRNA signature for prognosis of thymoma

**DOI:** 10.18632/aging.204108

**Published:** 2022-06-07

**Authors:** Bin Wang, He Xiao, Xin Yang, Ying Zeng, Zhimin Zhang, Rui Yang, Hang Chen, Chuan Chen, Junxia Chen

**Affiliations:** 1 Department of Cell Biology and Genetics, Chongqing Medical University, Chongqing 400016, China; 2 Department of Oncology, Daping Hospital, Army Medical University, Chongqing 400042, China; 3 Department of Pathology, Daping Hospital, Army Medical University, Chongqing 400042, China; 4 Department of Pathology, Chongqing General Hospital, University of Chinese Academy of Sciences, Chongqing 410309, China; 5 Department of Thoracic Surgery, The Third Medical Center of PLA General Hospital, Chongqing 100039, China

**Keywords:** immune-related microRNA signature, thymoma, biomarker-related recurrence risk score

## Abstract

Introduction: Immune microenvironment and microRNAs serve as common predictors for diagnosis and prognosis of tumors.

Methods: Expression of 122 genes and 126 microRNAs in thymoma was obtained from TCGA database. The proportion of tumor-infiltrating cells was calculated, and IMRS was constructed. TREM2hi score was calculated before functional enrichment analysis on gene sets.

Results: IMRS3, TREM2hi score, and CD8+ T lymphocyte abundance were significantly different among WHO classifications. WHO classification, Masaoka staging, and miR-130b-5p, miR-1307-3p, miR-425-5p, CD8, CD68, and CCL18 expression were prognostic factors for relapse-free survival and overall survival. IMRS3 upregulation polarized macrophages into M2, which rejected CD8+ T and other effector lymphocytes to promote thymoma malignant progression.

Conclusions: BRRS may present a novel immune-related microRNA signature for TET prognosis.

## INTRODUCTION

Thymic epithelial tumors (TETs) originate from thymic epithelial cells with a relatively low incidence of approximately 0.13/100,000 [1]. Currently, the recurrence and survival prognosis of TETs mainly rely on the World Health Organization (WHO) classification and Masaoka staging [2]. According to 2015 WHO classification, TETs can be divided into thymoma (types A, AB, B1, B2, and B3) and thymic carcinoma (type C) [3]. Types A, AB, B1, B2, and B3 TETs present a low malignancy, less recurrence, and good survival prognosis. In contrast, type C TETs are considered highly malignant and easy to relapse, together with a poor survival prognosis. In Masaoka staging, stages I-II are deemed to have a low risk of recurrence and a good survival prognosis, while stages III-IV present a high risk of recurrence and a poor survival prognosis. However, there are certain limitations to predicting recurrence and survival prognosis of TET patients as per the WHO classification and Masaoka staging system in a clinical setting. For example, recurrence has also been noted for type A, AB, or B1 thymomas with a low malignancy; early metastasis, among other factors, affects the prognosis of patients [4]. As demonstrated in recent studies, biomarkers that are related to the immune microenvironment are associated with the prognosis of TETs. Therefore, the identification of more accurate biomarkers carries tremendous clinical implications for predicting the prognosis of TETs [5].

Significant differences have been found in the expression of various microRNAs related to the immune microenvironment between normal and tumor cells. These differentially expressed microRNAs can act as proto-oncogenes or tumor suppressor genes by regulating different target genes, and their roles are closely related to the occurrence, development, treatment, and prognosis of tumors [6]. Tumor-associated macrophages are the most abundant immune cells in the tumor microenvironment. MicroRNAs regulate macrophage differentiation, functional polarization, and cellular crosstalk [7]. Abnormally expressed microRNAs can regulate the differentiation, proliferation, apoptosis, and function of immune cells, thereby affecting the occurrence and development of tumors [8]. Moreover, CCL18 is a marker of M2 macrophage subpopulations, and its elevated level in serum and tumors is associated with a poor prognosis [9]. The tumor microenvironment is an essential factor for tumor immune escape. The inhibition of immune response by the tumor microenvironment remains a major clinical concern during the administration of immunotherapy. The regulatory mechanism of microRNAs on immune cells in the tumor microenvironment can provide new targets for tumor immunotherapy [10]. However, few studies have been done on the impact of microRNAs on the immune microenvironment and the prognosis of TET patients. Here, we developed BRRS for TETs using microRNA-mRNA pairs and tumor-infiltrating immune cells from the TCGA dataset and performed validation in an independent cohort.

## RESULTS

### Identification of microRNAs influencing macrophage function on progression-free interval

In the TCGA thymoma datasets, PFI (progression-free interval) data and cell abundances inferred from EPIC (electronic Publication Information Center) were available for 97 tumor samples. Macrophages were the most pronounced cell population for the prognosis of PFI (Supplementary Table 2). Based on the macrophage abundance obtained by EPIC, 16 microRNAs and 850 genes were identified as significantly interacting with macrophages to affect PFI (Supplementary Table 3). A total of 41 microRNA-mRNA pairs were retrieved from the anamiR analysis (Supplementary Table 4). From the expression levels of these microRNAs supported by the microRNA datasets, LASSO (least absolute shrinkage and selection operator) returned a model containing four microRNAs with non-zero coefficients (Table 1). The baseline characteristics of the 119 samples with PFI data are shown in Supplementary Table 5 (sheet ‘Baseline of TCGA TYHM’). Although WHO histology (C vs. A-AB), hsa-miR-1307-3p, hsa-miR-425-5p, and TREM2^hi^ signature were significantly associated with PFI, the effects of IMRS3 and IMRS4 were more conspicuous (Supplementary Table 5, sheet ‘Univariate Cox regression’). Moreover, IMRS3 and IMRS4 were independent prognostic factors for PFI after adjustment for gender, age, WHO classification, Masaoka stage, and other individual biomarkers (Table 2). Taking HR values and 95% CIs into consideration, IMRS3 may be as reliable as IMRS4 in terms of the prognosis of PFI.

**Table 1:** MicroRNAs included into model resulting from LASSO.

**miRNAs**	**Coefficient**
hsa-miR-130b-5p	-0.440591996
hsa-miR-1307-3p	0.911975775
hsa-miR-425-5p	0.322839967
hsa-miR-425-3p	0.205127116

**Table 2:** Multivariate Cox regression for PFI illustrating independent prognosis of IMRS3 and IMRS4.

	**HR (95% CI)**	***P***	**HR (95% CI)**	***P***
Age	1.004 (0.969-1.041)	.827	1.005 (0.969-1.042)	.791
Gender (Male vs Female)	0.737 (0.288-1.888)	.525	0.720 (0.282-1.835)	.491
Myasthenia gravis (Yes vs No)	0.671 (0.225-2.003)	.474	0.660 (0.221-1.970)	.456
WHO histology (B1-B3 vs A-AB)	1.675 (0.503-5.581)	.401	1.789 (0.538-5.946)	.343
WHO histology (C vs A-AB)	1.039 (0.174-6.222)	.966	0.981 (0.159-6.050)	.983
Masaoka's stage (III-IVB vs I-II)	0.924 (0.265-3.225)	.901	0.858 (0.245-3.003)	.811
TREM2hi	1.334 (0.733-2.429)	.346	1.310 (0.714-2.405)	.383
IMRS4/IMRS3	2.633 (1.226-5.657)	.013	2.921 (1.273-6.701)	.011

### Characteristics of immune microenvironment in thymoma

**Figure 1:**
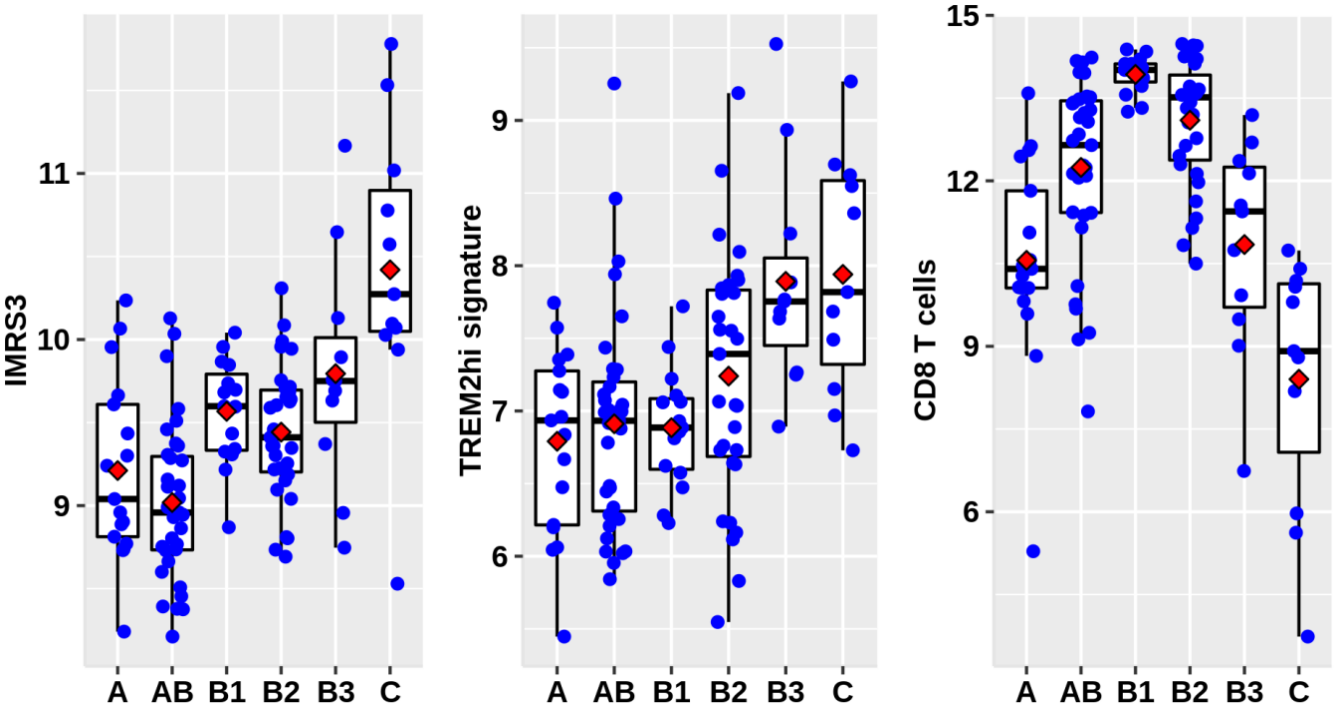
**Distribution of IMRS3, TREM2hi signature, and abundance of CD8+ T cells among the WHO classification.** Data were obtained from 122 thymoma gene expression values, as well as phenotype files and survival data all from the UCSC, expression matrix in the format of log2(x+1) transformed RSEM normalized counts.

**Figure 2:**
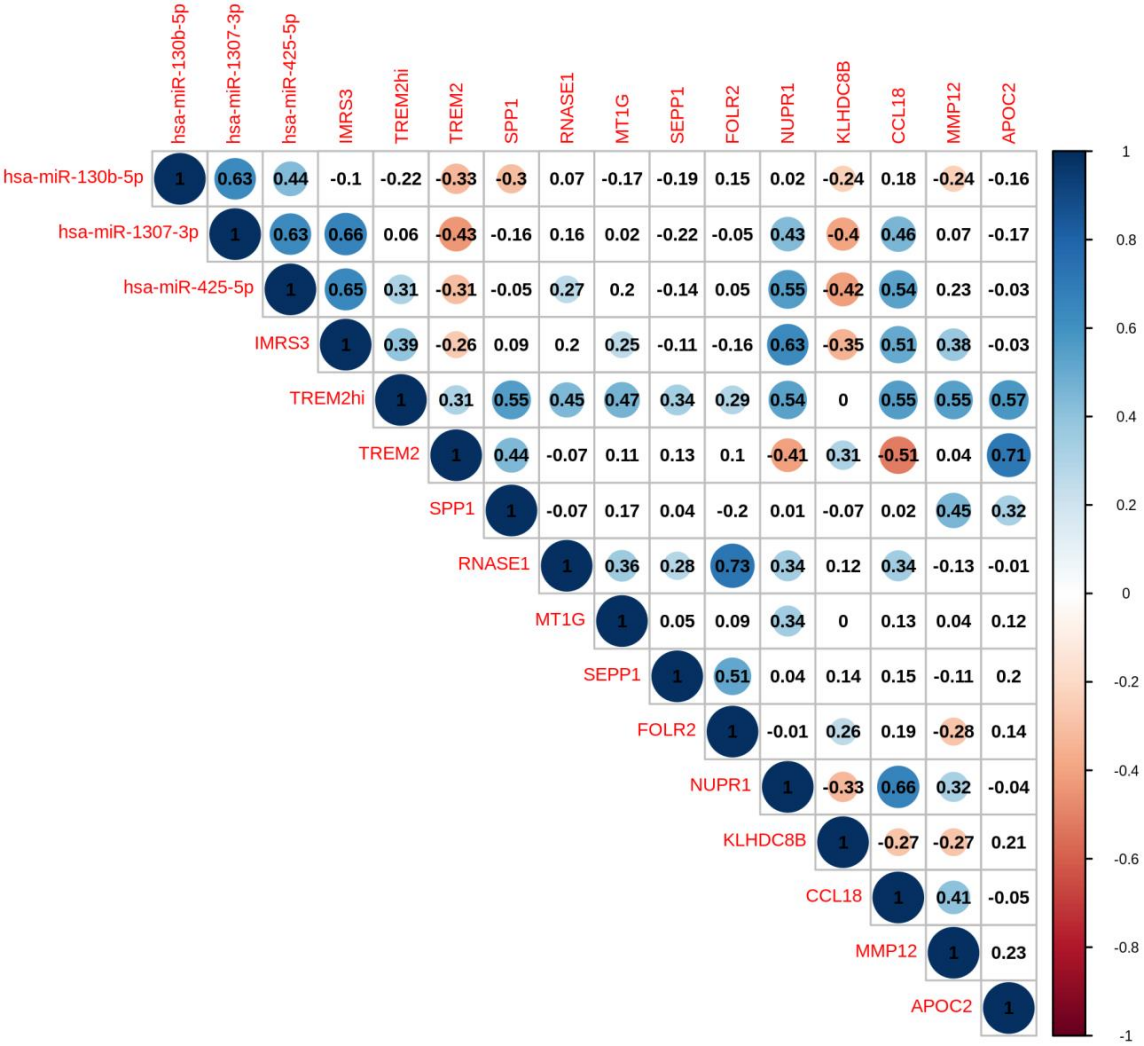
Pairwise correlation of TREMhi signature, microRNAs, and eleven genes in TREM2hi signature of 120 THYM samples.

As shown in Figure 1, there was a clear trend that IMRS3 and the TREM2^hi^ signature increased with the increase in the WHO grades. However, the abundance of CD8 T cells exhibited a parabolic change. Detailed pairwise comparisons are shown in Supplementary Table 6. Moreover, there was a significant positive correlation between IMRS3 and the TREM2^hi^ signature (Figure 2). In addition, CCL18 (Chemokine (C-C motif) ligand 18), one of the components of the TREM2^hi^ signature, was also highly positively correlated with miR-1307-3p, miR-425-5p, IMRS3, and TREM2^hi^ signature itself (Figure 2). Gene set enrichment analysis showed that tumors with high TREM2^hi^ signature had several critical immune pathways in addition to “interleukin-10 signaling”, such as “interferon-gamma signaling”, “antigen processing-cross presentation”, and “interferon-alpha/beta signaling” (Supplementary Table 7). These results indicated a complex regulatory network of infiltrating monocyte differentiation in thymoma tissues. Importantly, IMRS3 as well as macrophages, especially TREM2^hi^ macrophages, may be essential for thymoma progression.

### Correlation of microRNAs and immune-related biomarkers with clinical features

**Figure 3:**
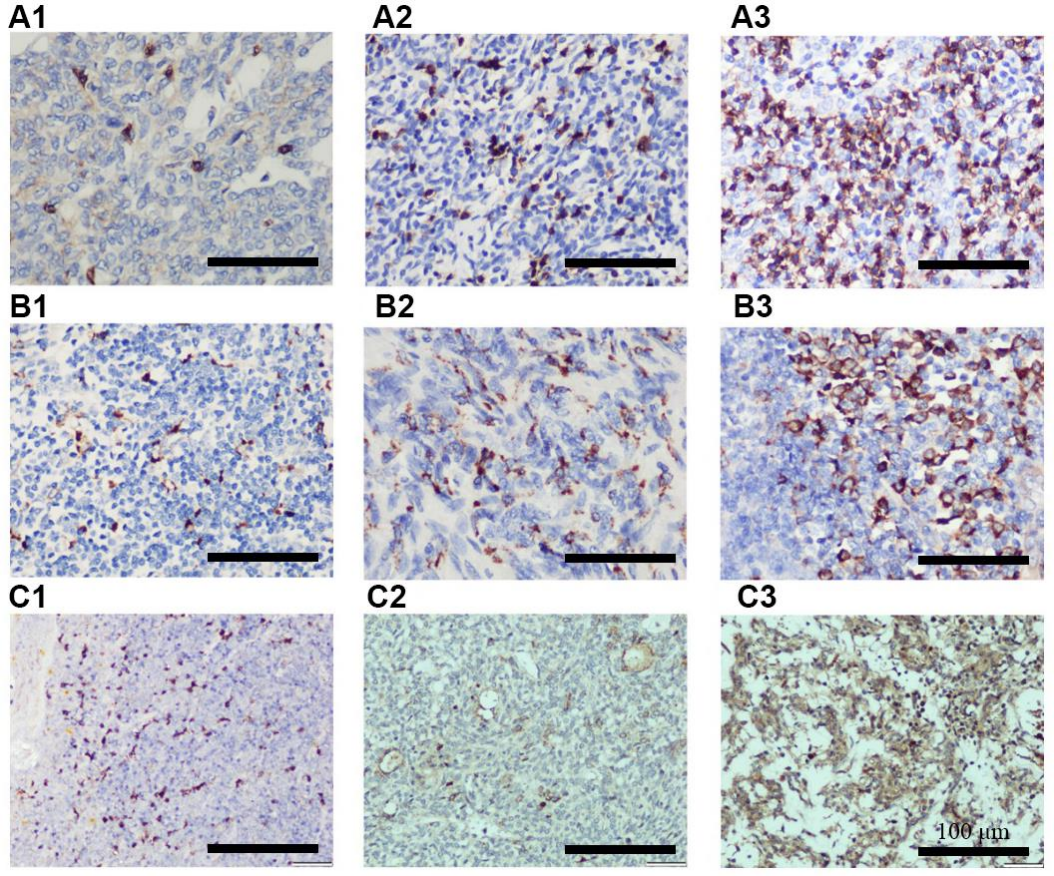
Representative IHC images showing different expression of CD8 (**A1**–**A3**), CD68 (**B1**–**B3**), and CCL18 (**C1**–**C3**). **A1**, **B1**, and **C1** indicates + IHC score; **A2**, **B2**, and **C2** indicates ++ IHC score; **A3**, **B3**, and **C3** indicates +++ IHC score. (n = 3 for **A**–**C** groups; scale bars, 200 μm).

To further explore the roles of miR-130b-5p, miR-1307-3p, miR-425-5p, and the TREM2^hi^ signature in determining RFS of thymoma, these biomarkers were evaluated in an independent cohort of 99 patients. Baseline characteristics of the cohort are shown in Supplementary Table 8. Consistent with the TCGA thymoma dataset, a significantly higher proportion of patients with WHO type C or Masaoka stage III-IV thymoma had increased expression of CD68 and CCL18 (Table 3). Representative IHC images of CD8, CD68, and CCL18 are shown in Figure 3. Furthermore, the expression levels of miR-130b-5p, miR-1307-3p, and miR-425-5p were all markedly increased in WHO type C compared with A-AB or B1-B3 types (Table 4). These results confirmed that miR-130b-5p, miR-1307-3p, and miR-425-5p might be involved in the progression of thymoma.

**Table 3:** Comparison of expression of CD8, CD68 and CCL18 in subtypes of WHO classification.

	**CD8**			**CD68**			**CCL18**		
	**Negative**	**Positive**	**χ^2^/*P***	**Negative**	**Positive**	**χ^2^/*P***	**Negative**	**Positive**	**χ^2^/*P***
Gender									
Female	12 (24.5)	37 (75.5)	1.081/0.299	33 (67.3)	16 (32.7)	1.846/0.174	29 (59.2)	20 (40.8)	0.842/0.359
Male	17 (34.0)	33 (66.0)		27 (54.0)	23 (46.0)		25 (50.0)	25 (50.0)	
Myasthenia gravis									
No	15 (28.8)	37 (71.2)	0.011/0.918	30 (57.7)	22 (42.3)	0.389/0.533	28 (53.8)	24 (46.2)	0.022/0.883
Yes	14 (29.8)	33 (70.2)		30 (63.8)	17 (36.2)		26 (55.3)	21 (44.7)	
WHO classification									
A-AB	1 (4.5)	21 (95.5)	34.372/<0.001	20 (90.9)	2 (9.1)	3.270/<0.001	17 (77.3)	5 (22.7)	21.541/<0.001
B1-B3	13 (21.7)	47 (78.3)		39 (65.0)	21 (35.0)		36 (60.0)	24 (40.0)	
C	15 (88.2)	2 (11.8)		1 (5.9)	16 (94.1)		1 (5.9)	16 (94.1)	
Masaoka's stage									
I-II	1 (1.7)	59 (98.3)	56.123/<0.001	54 (90.0)	6 (10.0)	55.118/<0.001	49 (81.7)	11 (18.3)	45.186/<0.001
III-IV	28 (71.8)	11 (28.2)		6 (15.4)	33 (84.6)		5 (12.8)	34 (87.2)	
Adjuvant radiotherapy									
No	26 (32.5)	54 (67.5)	2.070/<0.150	47 (58.8)	33 (41.3)	0.601/0.438	42 (52.5)	38 (47.5)	0.703/0.402
Yes	3 (15.8)	16 (84.2)		13 (68.4)	6 (31.6)		12 (63.2)	7 (36.8)	
Adjuvant chemotherapy									
No	28 (33.7)	55 (66.3)	3.655/0.056	48 (57.8)	35 (42.2)	1.656/0.198	42 (50.6)	41 (49.4)	3.220/0.073
Yes	1 (6.3)	15 (93.8)		12 (75.0)	4 (25.0)		12 (75.0)	4 (25.0)	

**Table 4:** Comparison of expression level of microRNAs among WHO classification, Masaoka’s stage and CD8, CD68 and CCL18 lowly and highly expressed subgroups determined by immunohistochemistry.

	**miR-130b-5p**	**miR-425-5p**	**miR-1307-3p**	**BRRS**
WHO classification				
A-AB	0.085 (-0.610-0.261)	0.063 (-0.636-0.179)	0.329 (-0.260-0.539)	-1.049 (-1.733-(0.887))
B1-B3	0.056 (-0.388-0.417)	-0.016 (-0.341-0.439)	0.391 (-0.093-0.783)	-1.140 (-1.452-0.700)
C	1.049 (0.775-1.464)	1.217 (0.993-1.342)	1.627 (1.236-1.718)	2.479 (2.266-2.599)
Kruskal-Wallis *P*	<0.001	<0.001	<0.001	<0.001
B1-B3 vs C adjusted *P*	<0.001	<0.001	<0.001	<0.001
B1-B3 vs A-AB adjusted *P*	1.000	0.965	0.800	0.965
C vs A-AB adjusted *P*	<0.001	<0.001	<0.001	<0.001
Masaoka's stage				
I-II	-0.174 (-0.587-0.188)	-0.152 (-0.545-0.132)	0.194 (-0.326-0.466)	-1.271 (-1.646-(-0.916))
III-IV	0.705 (0.314-1.163)	0.752 (0.407-1.217)	1.096 (0.815-1.599)	1.962 (0.707-2.480)
Mann-Whitney *P*	<0.001	<0.001	<0.001	<0.001
CD8 IHC				
Low	0.832 (0.552-1.256)	1.042 (0.618-1.230)	1.316 (0.936-1.633)	2.313 (1.909-2.492)
High	-0.116 (-0.561-0.189)	-0.112 (-0.502-0.179)	0.238 (-0.260-0.565)	-1.233 (-1.605-(-0.887))
Mann-Whitney *P*	<0.001	<0.001	<0.001	<0.001
CD68 IHC				
Low	-0.221 (-0.617-0.155)	-0.183 (-0.545-0.063)	0.139 (-0.326-0.415)	-1.302 (-1.646-(-1.026))
High	0.716 (0.475-1.163)	0.752 (0.407-1.217)	1.096 (0.815-1.599)	1.962 (0.693-2.480)
Mann-Whitney *P*	<0.001	<0.001	<0.001	<0.001
CCL18 IHC				
Low	-0.257 (-0.646-0.111)	-0.277 (-0.564-0.011)	0.054 (-0.362-0.336)	-1.391 (-1.664-(-1.116))
High	0.608 (0.314-1.049)	0.618 (0.280-1.141)	0.936 (0.716-1.485)	1.882 (0.643-2.407)
Mann-Whitney *P*	<0.001	<0.001	<0.001	<0.001

### Validation of biomarker-related recurrence risk scores

**Figure 4:**
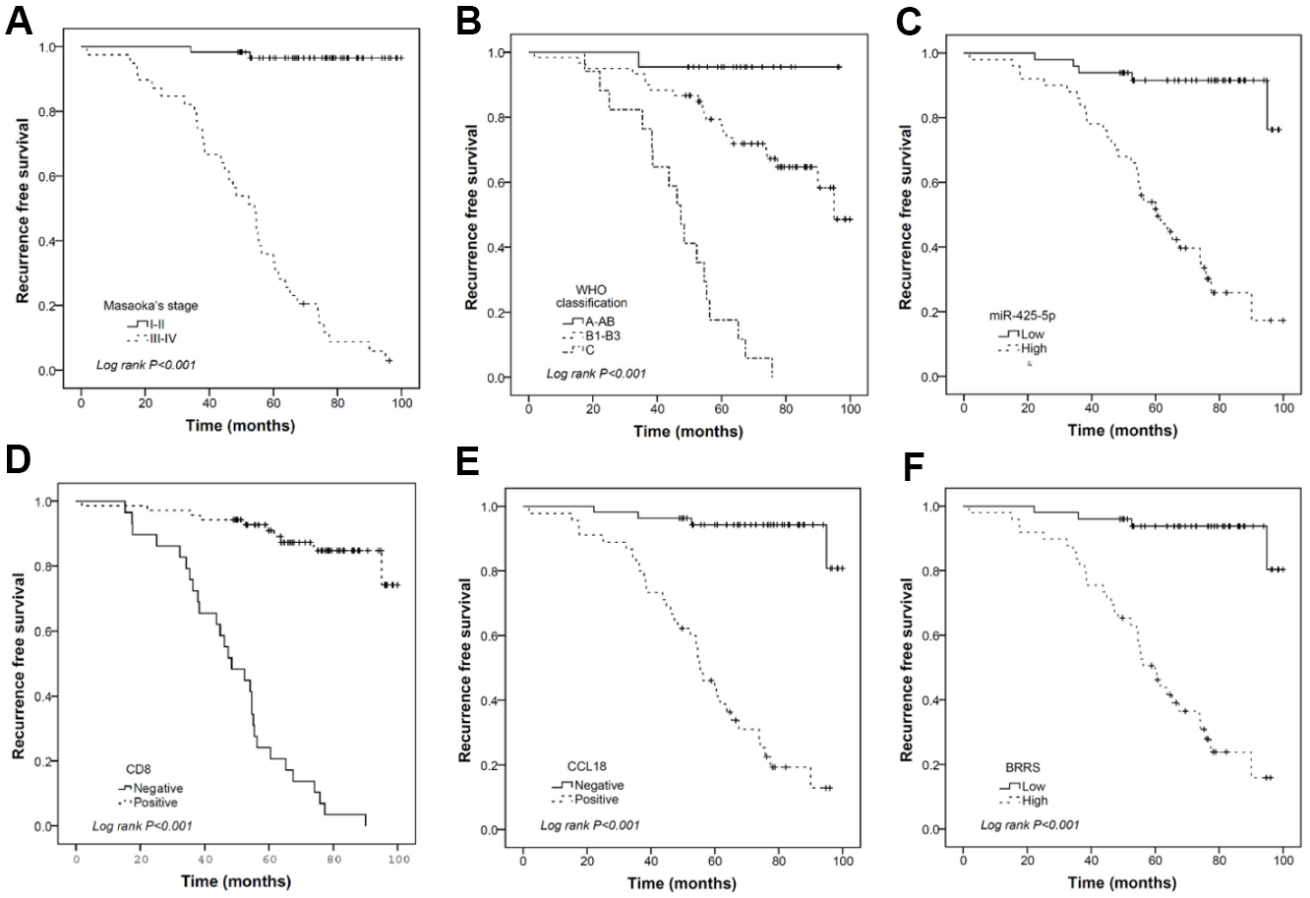
Kaplan-Meier curves illustrating the prognostic effects of Masaoka stage (**A**), WHO classification (**B**), miR-425-5p (**C**), CD8 (**D**), CCL18 (**E**), and BRRS (**F**) categorized by corresponding median values. (n = 120 THYM samples).

**Figure 5:**
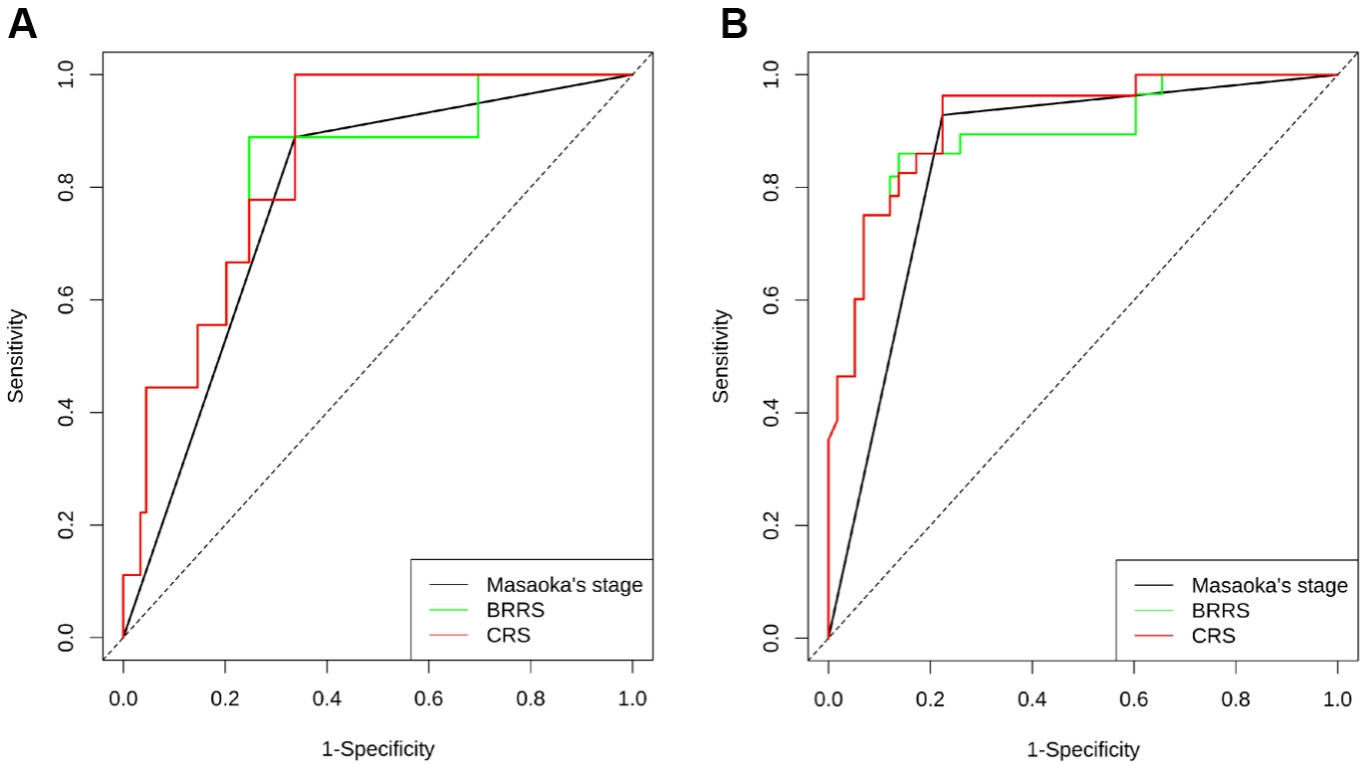
Time-dependent ROC illustrating the accuracy of Masaoka stage, BRRS, and CRS for predicting the RFS at 3 years (**A**) and 5 years (**B**). (n = 120 THYM samples).

As shown in Supplementary Table 8, all the six biomarkers (miR-130b-5p, miR-1307-3p, miR-425-5p, CD8, CD68, and CCL18) were strongly associated with RFS. However, forward stepwise Cox regression revealed that only miR-425-5p, CD8, and CCL18 were independent prognostic factors for RFS in our cohort. BRRS was significantly greater in WHO type C than in types A-AB or B1-B3 (Table 4) and was positively associated with the risks of recurrence and death as revealed by the univariate Cox regression (HR=2.718, 95% CI: 2.068-3.573, *P*<0.001; HR=2.779, 95% CI: 1.700-4.544, *P*<0.001). Multivariate Cox regression showed that BRRS remained significant for RFS but not for OS after being adjusted for gender, age, WHO classification, Masaoka stage, adjuvant chemotherapy, and adjuvant radiotherapy (Table 5 and Figure 4). Time-dependent ROC analysis revealed that BRRS predicted 5-year RFS with better accuracy than miR-425-5p or CCL18 alone (Table 6 and Figure 5). Moreover, the AUC of BRRS for the 5-year RFS was significantly higher than that of the Masaoka stage (AUC: 92.0 vs. 85.2, *P*=0.007). However, the difference in AUC for OS between CRS and Masaoka stage did not reach significance (Table 6).

**Table 5:** Multivariate Cox regression for RFS and OS in an independent thymoma cohort.

	**RFS**		**OS**	
	**HR (95% C.I.)**	***P***	**HR (95% C.I.)**	***P***
Age	1.024 (1.002-1.047)	.031	1.037 (0.997-1.079)	.069
Gender (Male vs Female)	1.387 (0.672-2.862)	.377	0.517 (0.160-1.666)	.269
Myasthenia gravis (Yes vs No)	1.592 (0.788-3.216)	.195	1.353 (0.428-4.274)	.607
WHO classification (C vs A-B3)	0.807 (0.371-1.754)	.588	1.810 (0.437-7.501)	.413
Masaoka's stage (III-IV vs I-II)	14.115 (2.740-72.723)	.002	1.739 (0.087-34.878)	.718
Adjuvant radiotherapy (Yes vs No)	3.450 (1.184-10.058)	.023	0.954 (0.073-12.457)	.971
Adjuvant chemotherapy (Yes vs No)	0.850 (0.235-3.070)	.804	1.34×10^-5^ (-)	.979
BRRS	2.212 (1.403-3.486)	.001	2.239 (0.886-5.662)	.089

The BRRS parameters in the table combine multiple biomarkers (including miR-130b-5p, miR-1307-3p, miR-425-5p, CD8, CD68 and CCL18).

**Table 6:** Time-dependent ROC analysis showing various accuracies for predicting 3- and 5-year RFS and OS.

**RFS**	**Time-dependent AUC (3 year)**	**Time-dependent AUC (5 year)**	**Compared with BRRS at 3 year or 5 year**	**Compared with CRS at 3 year or 5 year**
miR-425-5p	0.783 (0.613-0.953)	0.875 (0.791-0.960)	0.556/0.397	
CD8	0.765 (0.622-0.901)	0.832 (0.745-0.920)	0.479/0.044	
CCL18	0.737 (0.621-0.852)	0.792 (0.709-0.875)	0.064/1.183×10-4	
BRRS	0.815 (0.674-0.956)	0.893 (0.816-0.970)		0.631/0.275
Masaoka's stage	0.776 (0.662-0.890)	0.852 (0.780-0.924)		0.261/0.007
CRS	0.845 (0.748-0.942)	0.920 (0.861-0.980)		
OS				
miR-425-5p	0.806 (0.724-0.887)	0.790 (0.592-0.987)	0.334/0.415	
CD8	0.693 (0.421-0.965)	0.710 (0.509-0.911)	0.507/0.298	
CCL18	0.781 (0.731-0.831)	0.697 (0.521-0.874)	0.951/0.067	
BRRS	0.771 (0.678-0.863)	0.776 (0.580-0.972)		0.686/0.703
Masaoka's stage	0.813 (0.764-0.861)	0.726 (0.550-0.902)		0.465/0.349
CRS	0.774 (0.684-0.864)	0.773 (0.573-0.973)		

## DISCUSSION

TETs arise from thymic epithelial cells with a low morbidity rate. However, few studies have been done on the treatment and prognosis of those patients presenting with TETs. Here, we developed BRRS for TETs using microRNA-mRNA pairs and tumor-infiltrating immune cells from the TCGA dataset. Our analysis of 97 tumor samples from the TCGA thymoma datasets indicated that macrophage infiltration abundance was the most pronounced prognostic factor for PFI. A total of 41 microRNA-mRNA pairs were further analyzed to obtain 4 microRNAs responsible for biomarkers of PFI. Then, we analyzed the clinical features and found that the prognostic indicators consisting of microRNAs were superior to the current clinical staging.

IMRS3 and CD8+ T cells were found related to the TREM2^hi^ score. In addition, we validated miR-435-5p, CD8, and CCL18 as independent predictors of RFS. In the 99 cases, BRRS was significantly related to the recurrence risk and death risk. Notably, after adjusting for gender, age, myasthenia gravis, Masaoka staging, WHO classification, radiotherapy, and chemotherapy, BRRS remained an independent prognostic factor for RFS. The tumor microenvironment has important interactions with cancer cells to determine outcomes, and especially immune-related microRNAs are important biomarkers for prognosis and treatment response [11]. The immune-related microRNA signature was strongly correlated with the immune microenvironment and clinical outcomes of hepatocellular carcinoma [12]. The expression of immune-related microRNAs was associated with the role of the tumor immune microenvironment in immunosuppressive therapy [13]. In this study, BRRS, independent of Masaoka staging, had a significant regression contribution to RFS, suggesting that BRRS may serve as a promising signature in addition to the current clinical prognostic indicators.

Additionally, we validated that IMRS3 is related to TREM2^hi^ and acts as a prognostic factor superior to the current clinical staging. Besides, IMRS of macrophages was significantly correlated with the thymoma PFI collected from the TCGA dataset. Immune-related genes and microRNAs have been widely studied, and they hold significate research value for cancer prognosis. Immune-related genes can effectively predict the clinical outcome of hepatocellular carcinoma treatment and assess the clinical status of the tumor [14]. Guo et al. developed an immune-based prognostic model for renal cell carcinoma, validating that the immune-related microRNA signature is a feasible and reliable prognostic model [15]. Immune-related microRNAs are non-invasive biomarkers of clinical outcomes and modulate the immunosuppressive tumor microenvironment in metastatic melanoma [11]. Immune-related microRNAs regulate immune cells directly [15] or may modulate the immune microenvironment through exosomes. Unlike the study in which prognostic markers were constructed with potential microRNAs, the present study applied a more indirect statistical-based approach to screen microRNAs that may modulate macrophage function. We successfully validated that miR-130b-5p, miR-1307-3p, and miR-425-5p in tumors were significantly related to the WHO classification, and their increased expression levels may be associated with the development of immunosuppressive microenvironment in thymoma. All IMRS, WHO classification, and Masaoka staging were prognostic factors for RFS and OS. Therefore, we believe that immune-related microRNAs and IMRS may be vital contributors in predicting the prognosis of thymoma. In this sense, the immune-related microRNAs we identified differ from the “immune-related microRNAs” defined by microRNA species that directly affect gene regulation in immune cells.

In the 120 thymomas, IMRS3 and TREM2^hi^ scores were found significantly positively correlated, while TREM2^hi^ was negatively correlated with CD8+ T lymphocytes. Thymoma led to polarization of M2 macrophages due to high expression of IMRS3, which rejected CD8 T and other effector lymphocytes and promoted the malignant progression of thymoma. The high TREM2^hi^ score expression group was mainly characterized by immunoregulatory pathways such as the IL-10 signaling pathway and IFN-γ pathway. The expression of hsa-microRNA-1307-3p and hsa-miR-425-5p, which were significantly correlated with PFI in IMRS3, had a strong positive correlation with CCL18. Hence, we proposed that immune-related microRNAs may be a biomarker for the immunosuppressive tumor microenvironment through modulating IL-10 or IFN-γ-mediated signaling pathway. Immune-related microRNAs regulate immunoregulatory molecules, which mediate immune escape from cancer and result in a poor prognosis. Immunoregulatory microRNAs, as a novel mechanism, can reverse immune escape from tumors [16]. Proinflammatory cytokines (interleukin and tumor necrosis factor-mediated signaling pathways) are regulated by the expression of immune-related microRNAs and associated with colorectal tumor progression, which can be considered as biomarkers of colorectal cancer (immune microenvironment of colorectal tumors: involvement of immune genes and microRNAs belonging to the TH17 pathway). Moreover, IMRS3 expression was significantly associated with CD8 expression in the IHC data of our clinical samples. Therefore, we believe that immune-related microRNAs can act as an accurate prognostic marker for thymoma in the future.

CD8 is a marker of killer T lymphocytes and CD68 is a marker of tumour-associated macrophages (TAM). CCL18 is primarily produced and secreted by the innate immune system (including TAM) and affects primarily the adaptive immune system (lymphocytes). These three interact to ultimately recruit Th2 and regulatory T cells, which mediate immunosuppressive effects; and secrete a variety of cytokines, growth factors (e.g. EGF, VEGF, PDGF, FGF and TGFβ), which interact with and influence multiple cell types in the tumor microenvironment and, in turn, mediate complex effects. Thus, the triad of CD8, CD68 and CCL18 clearly influences tumor prognosis, which corroborates our findings in the cox regression analysis.

Our study still has some limitations. First, we used the MCPcounter method to analyze immune cell abundance, which simply utilized the geometric means of the gene tag expression values of 10 immune cells to indirectly measure the abundance of infiltration, which may not represent the physical meaning of the actual infiltration ratio. Second, although the immune-related RNAs we proposed show great predictive value for the prognosis of thymoma, more in-depth studies are highly warranted to validate this finding. In addition, the immune-related microRNAs we identified in the present study need to use scRNAseq data to determine in a more accurate way whether expression of thymoma epithelial cells and microenvironmental macrophages via exosomes and other methods lead to an immunosuppressive environment. The specific mechanism needs to be further investigated. Despite these shortcomings, our research still has important scientific implications. For the first time, we propose that immune-related microRNAs play a critical role in thymoma prognosis and have great potential for clinical use.

Immune-related microRNAs may serve as essential biomarkers for predicting the prognosis of thymoma, and they may affect the prognosis by modulating the immune microenvironment. Our efforts provide a promising approach for accurate judgment of thymoma progression, leading to a new research interest that immune-related microRNAs that mediate the immune microenvironment may contribute to poor prognosis and even pathogenesis of thymoma.

## MATERIALS AND METHODS

### Dataset retrieval and preprocessing

Gene expression values, as well as phenotype files and survival data for 122 thymomas, were downloaded from UCSC (https://xenabrowser.net/datapages/), and the expression matrices were in a format of log2(x+1)-transformed RSEM normalized counts. Genes with 0 count more than 20% of the samples were filtered, and 16,552 genes were included. Additionally, we also downloaded microRNAs expression matrices for 126 thymomas from UCSC in log2(RPM+1) format, and 411 microRNAs were available for analysis after filtering the microRNAs with NA-containing expression values. Accession ID numbers of microRNAs were transformed with mature microRNA names using the miRBaseConverter package [17], and a total of 408 annotated microRNAs were finally included.

### Evaluation of the abundance of tumor-infiltrating immune cells

The abundance of 8, 22, and 10 immune cells was analyzed using the EPIC, CIBERSORTx, and MCPcounter methods, respectively [18, 19]. For EPIC, count values were used as imported data to obtain the abundance of 8 cell populations (including CD4 T cells, CD8 T cells, NK cells, and macrophages) via deconvolution. Similar to EPIC, CIBERSORTx also utilized deconvolution to obtain 22 immune cell abundances. In the MCPcounter process, the infiltration abundance of 10 immune cells was indirectly measured with the geometric mean of their tag expression values.

### Identification of microRNA-mRNA pairs influencing macrophage function on relapse-free survival

In order to screen out the microRNAs and gene mRNAs that may indirectly affect RFS by affecting tumor immune cell function, a similar method for constructing T cell depletion signatures proposed by Jiang [20] was used. Subsequently, a prognostic microRNA-mRNA regulatory network for the identification method was constructed using the microRNA-mRNA target genes.

By univariate Cox regression, “macrophage” abundance obtained by EPIC was found to be a significant prognostic factor for PFI (β = 90.08, *P* = 0.0113). Given this, regression analysis of the 408 microRNAs was performed using macrophage abundance, microRNA expression values, and Cox regression of interaction terms between the two, and mRNA expression values of the 16,552 genes underwent Cox regression in a similar manner. MicroRNA species and mRNA genes with macrophage abundance, expression values, and interaction term regression coefficients Wald *P* < 0.05 were extracted as candidate genes.

The correlation of the expression values of these candidate genes was further analyzed with the R package anamiR function “negativecor” using the Pearson correlation coefficient method. MicroRNA-mRNA target gene pairs with r < −0.5 were included in the “databasesupport” function [21] to obtain microRNA-mRNA target gene pairs predicted by the miRNA database, and the microRNAs whose interaction with mRNAs was supported by at least three datasets were finally selected as candidates for weighted linear modeling. Penalized Cox regression was performed using LASSO to obtain a PFI prognostic signature containing four microRNA species, which was named the immune-related microRNAs score (IMRS). In order to make validation more feasible in a clinical setting, a reduced model incorporating the first three microRNAs (IMRS3) was calculated in comparison to the entire model (IMRS4) in terms of PFI prognostic performance. Fivefold cross-validation was conducted to select the lamda using the function cv.glmnet [22]. IMRS was calculated by summing the expression levels of microRNAs weighted with the corresponding non-zero coefficients.

### Characterization of the immune microenvironment highly infiltrated by M2 macrophages

To further reveal the correlation of IMRS with M2 macrophages and T cells, a gene signature peculiar to TREM2^hi^ macrophages was utilized [23], which consists of 11 genes (TREM2, SPP1, RNASE1, MT1G, SEPP1, FOLR2, NUPR1, KLHDC8B, CCL18, MMP12, and APOC2). The mean expression value of the 11 genes was taken as the TREM2^hi^ score.

The correlation between the 11 genes, TREM2^hi^ score, infiltrating immune cell abundance, and IMRS was analyzed using the Pearson method. In order to further explore the effect of TREM2^hi^ score on tumor immune microenvironment, 120 thymoma samples were divided by the median TREM2^hi^ score into high and low expression groups, and differentially expressed genes were obtained using “limma” package “voom” and “lmFit”. The enrichment analysis of the Reactome gene set was done using the clusterProfiler package “gesPathway” function with the *t* as the test variable [24], and a corrected *P* < 0.05 was taken as the significantly enriched pathway.

### Patients

To explore the correlation between the expression of the three candidate microRNAs, immune cells, and RFS, 99 patients with histologically confirmed thymoma or thymic carcinoma who were admitted into the Department of Thoracic Surgery at Daping Hospital of the Third Military Medical University between January 2013 and April 2017 were enrolled in this study. Those with malignancies were excluded. The tumors were classified according to the 2015 WHO criteria and then staged as per the modified Masaoka staging system. Approval for this study was gained from the Medical Ethics Committee at the hospital (approval no. 2021-100), and written informed consent was obtained from all patients prior to enrolment. Patient information, including age, gender, histological findings, presence of myasthenia gravis, Masaoka stage, WHO histological type, and follow-up information, was collected from clinical records and by questionnaires.

### Quantitative RT-PCR (qRT-PCR)

We extracted total RNA from the 99 formalin-fixed, paraffin-embedded specimens with the Tiangen RNAprep Pure FFPE Kit (TIANGEN, Beijing, China, Category Number: DP439), detected the RNA concentration and purity with Nano Drop® 2000 (Thermo Fisher Scientific, CA, USA), reversely transcribed cDNA with the HiScript 1st Strand cDNA Synthesis Kit, and then conducted quantitative PCR reactions using AceQ qPCR SYBR Green Master Mix (without ROX) (Vazyme Biotech Co., Ltd, Nanjing, China) and Bio-Rad CFX96 system (Bio-Rad Laboratories, Inc., China). Each sample was assayed in triplicate, and U6 snRNA was used for normalization. The expression level of individual microRNA was determined by the 2^-ΔΔCT^ method (ΔCT = CT_microRNA_ – CT_U6RNA_), and one patient specimen was randomly selected as a reference sample. The primers used in this study are shown in Supplementary Table 1.

### Immunohistochemistry

For immunohistochemistry analysis, the paraffin-embedded tissue blocks were cut into sections at 3 μm thickness, and human CCL18 polyclonal antibodies (catalog no. ab233099, Cambridge, MA, USA) as well as CD68 and CD8 primary antibodies (catalog nos. ZM-0060 and ZA-0508, Zhong Shan Golden Bridge Biological Technology, Beijing, China) were used. Subsequently, immunostaining was done with the SPlink Detection kit (Biotin-Streptavidin HRP Detection System; catalog no. SP-9001; Zhong Shan Golden Bridge Biological Technology, Beijing, China) according to the manufacturer’s protocol. Slides were examined by two independent pathologists (Department of Pathology, Daping Hospital, Army Medical University) who had no prior knowledge of the clinical and pathological parameters. The staining intensity was classified as follows: grade 0: no staining; grade 1: definite but weak staining; grade 2: moderate staining; grade 3: strong staining. Positive cells was counted at ×400 magnification (range, 0-100%) in five random microscopic fields and categorized as follows: grade 0: no staining; grade 1: ≤10%; grade 2: 11%-50%; grade 3: 51%-75%; grade 4: >75%. The final scores were calculated by multiplying the proportion score by the intensity score, which were classified into 4 grades: 0, negative (-); 1-4, (+); 5-8 (++); 9-12 (+++). Samples that were ++ or +++ were defined as highly expressed and the rest as lowly expressed.

### Statistical analysis

All microRNA expression levels were transformed logarithmically and expressed as median values and interquartile ranges. The differences in microRNA expression among the WHO classifications, between Masaoka stage I-II and Masaoka stage III-IV subgroups, or between high and low expression subgroups of CD8, CD68, and CCL18 were evaluated by Kruskal-Wallis test or Mann-Whitney U test. The χ^2^ test or Fisher’s exact probability was applied to analyze the correlation between CD8, CD68, CCL18, and clinicopathological factors. The RFS of different subgroups was compared using the Kaplan-Meier method and the log-rank test, and prognostic factors for RFS and OS were identified in univariate and multivariate Cox proportional hazard regression models. In particular, a likelihood ratio test was used to determine whether a covariate was significantly entered into the regression model. Two weighted linear models were developed, one of which was based on the biomarkers in question (miR-130b-5p, miR-1307-3p, miR-425-5p, CD8, CD68, and CCL18), i.e., BRRS, and the other was based on a combination of BRRS and clinical features, i.e., combined risk scores (CRS). The predictive efficiency of Masaoka staging, BRRS, CRS, and other individual biomarkers for 3- and 5-year RFS and OS was determined with time-dependent ROC curve analysis by using the function “timeROC” and the function “compare” implemented in the R package. All other statistical analyses were performed using the SPSS 17.0 software (IBM SPSS, Chicago, IL, USA). All tests were bilateral, and *P* < 0.05 was considered statistically significant.

### Ethical disclosure

The study was authorized by the ethics Committee of Army Medical Center of PLA Approval of Medical Research Involving People Ethical Ratification NO:2021 (100).

## Supplementary Material

Supplementary Table 1

Supplementary Table 2

Supplementary Table 3

Supplementary Table 4

Supplementary Table 5

Supplementary Table 6

Supplementary Table 7

Supplementary Table 8
