# Supplementary material for: A novel immune-related microRNA signature for prognosis of thymoma
Source: Aging (Albany NY). 2022 Jun 7;14(11):4739–54. doi: 10.18632/aging.204108 (PMC9217705; doi:10.18632/aging.204108)
Supplement: Supplementary Table 1 [file aging-14-204108-s001.pdf]

Supplementary Table 1. Primers used in qTR-PCR.

Name	Sequence (5'-3')	Content
QRT290-miR130b-5p	GTCGTATCCAGTGCAGGGTCCGAGGTATTCGCACTGGATAC GACGTAGTG	Stem-loop sequence
QRT290-miR130b-F	CGCGACTCTTTCCCTGTTG	Forward Primer
QRT290-miR1307-3p	GTCGTATCCAGTGCAGGGTCCGAGGTATTCGCACTGGATAC GACCACGAC	Stem-loop sequence
QRT290-miR1307-F	ACTCGGCGTGGCGTCG	Forward Primer
QRT290-miR425-5p	GTCGTATCCAGTGCAGGGTCCGAGGTATTCGCACTGGATAC GACTCAACG	Stem-loop sequence
QRT290-miR425-F	GCGAATGACACGATCACTCC	Forward Primer
miRNA-R	AGTGCAGGGTCCGAGGTATT	Common Reverse Primer
Homo-U6-F	CTCGCTTCGGCAGCACA	Forward Primer
Homo-U6-R	AACGCTTCACGAATTTGCGT	Reverse Primer
