# Supplementary material for: A novel immune-related microRNA signature for prognosis of thymoma
Source: Aging (Albany NY). 2022 Jun 7;14(11):4739–54. doi: 10.18632/aging.204108 (PMC9217705; doi:10.18632/aging.204108)
Supplement: Supplementary Table 5 [file aging-14-204108-s005.pdf]

Supplementary Table 5. Baseline characteristics of 119 patients in TCGA thymoma dataset and univariate Cox regression for PFI.

		n (%)/Median (IQR)
Gender	Female	56 (47.1)
	Male	63 (52.9)
Myasthenia gravis	No	85 (71.4)
	Yes	34 (28.6)
Histology	A-AB	51 (42.9)
	B1-B3	57 (47.9)
	C	11 (9.2)
Masaoka's stage	I-II	98 (82.4)
	III-IVB	21 (17.6)
PFI	Censored	98 (82.4)
	Event	21 (17.6)
Age		60 (48-69)
hsa-miR-130b-5p		5.992 (4.6143-6.7622)
hsa-miR-1307-3p		10.5194 (10.0016-10.8819)
hsa-miR-425-5p		7.5604 (6.8810-8.0832)
hsa-miR-425-3p		3.6912 (3.2567-4.1300)
IMRS3		9.3606 (8.9454-9.7572)
IMRS4		10.1972 (9.6523-10.6129)
TREM2hi		7.0596 (6.6221-7.6845)

	β	SE	Wald	*P*	HR (95% CI)
Age	-0.005	0.016	0.081	0.777	0.995 (0.965-1.027)
Gender (Male vs Female)	-0.32	0.438	0.533	0.465	0.726 (0.308-1.714)
Myasthenia gravis (Yes vs No)	-0.313	0.515	0.37	0.543	0.731 (0.267-2.005)
WHO histology (B1-B3 vs A-AB)	0.915	0.574	2.537	0.111	2.496 (0.810-7.690)
WHO histology (C vs A-AB)	1.703	0.711	5.736	0.017	5.491 (1.363-22.131)
Masaoka's stage (III-IVB vs I-II)	0.34	0.562	0.367	0.545	1.405 (0.467-4.226)
has-miR-130b-5p	-0.149	0.165	0.818	0.366	0.861 (0.623-1.191)
has-miR-1307-3p	0.946	0.401	5.562	0.018	2.575 (1.173-5.650)
has-miR-425-5p	0.677	0.258	6.876	0.009	1.967 (1.186-3.262)
has-miR425-3p	0.593	0.398	2.222	0.136	1.810 (0.830-3.946)
IMRS3	1.179	0.275	18.414	<0.001	3.251 (1.897-5.570)
IMRS4	1.115	0.265	17.683	<0.001	3.048 (1.813-5.125)
TREM2hi	0.601	0.236	6.454	0.011	1.823 (1.147-2.898)
