# Supplementary material for: A novel immune-related microRNA signature for prognosis of thymoma
Source: Aging (Albany NY). 2022 Jun 7;14(11):4739–54. doi: 10.18632/aging.204108 (PMC9217705; doi:10.18632/aging.204108)
Supplement: Supplementary Table 8 [file aging-14-204108-s008.pdf]

Supplementary Table 8. Baseline characteristics of 99 thymoma patients in the validation set, univariate Cox regression and the final model from the forward stepwise Cox regression.

		n (%)
Gender	Female	49 (49.5)
	Male	50 (50.5)
Myasthenia gravis	No	52 (52.5)
	Yes	47 (47.5)
WHO classification	A-AB	22 (22.2)
	B1-B3	60 (60.6)
	C	17 (17.2)
Masaoka's stage	I-II	60 (60.6)
	III-IV	39 (39.4)
Adjuvant radiotherapy	No	80 (80.8)
	Yes	19 (19.2)
Adjuvant chemotherapy	No	83 (83.8)
	Yes	16 (16.2)
RFS	Censored	60 (60.6)
	Event	39 (39.4)
OS	Censored	85 (85.9)
	Event	14 (14.1)
CD8	Low	29 (29.3)
	High	70 (70.7)
CD68	Low	60 (60.6)
	High	39 (39.4)
CCL18	Low	54 (54.5)
	High	45 (45.5)
Age		52 (43-63)
miR-130b-5p		0.164 (-0.335-0.552)
miR-425-5p		0.124 (-0.321-0.563)
miR-1307-3p		0.468 (0.000-0.897)
BRRS		-0.924 (-1.43-1.74)

	RFS		OS	
	HR (95% C.I.)	*P*	HR (95% C.I.)	*P*
Age	1.003 (0.981-1.024)	0.816	1.030 (0.990-1.071)	0.142
Gender (Male vs Female)	1.336 (0.709-2.516)	0.371	0.687 (0.238-1.984)	0.488
Myasthenia gravis (Yes vs No)	0.955 (0.508-1.796)	0.887	0.782 (0.271-2.262)	0.65
WHO classification (C vs A-B3)	8.380 (4.239-16.568)	<0.001	9.819 (3.285-29.350)	<0.001
Masaoka's stage (III-IV vs I-II)	52.370 (12.546-218.60)	<0.001	21.272 (2.777-162.96)	0.003
Adjuvant radiotherapy (Yes vs No)	1.195 (0.567-2.522)	0.639	0.250 (0.033-1.918)	0.182
Adjuvant chemotherapy (Yes vs No)	0.555 (0.197-1.565)	0.266	0.037 (7.393E10-5-18.846)	0.3
miR-130b-5p	4.706 (3.090-7.167)	<0.001	4.184 (2.348-7.454)	<0.001
miR-425-5p	6.441 (4.010-10.346)	<0.001	5.269 (2.798-9.923)	<0.001
miR-1307-3p	4.356 (2.945-6.441)	<0.001	4.281 (2.369-7.739)	<0.001
CD8 (High vs Low)	0.062 (0.029-0.134)	<0.001	0.056 (0.012-0.249)	<0.001
CD68 (High vs Low)	16.282 (6.316-41.978)	<0.001	21.548 (2.816-164.86)	0.003
CCL18 (High vs Low)	17.158 (6.043-48.722)	<0.001	17.528 (2.290-134.16)	0.006
BRRS	2.718 (2.068-3.573)	<0.001	2.779 (1.700-4.544)	<0.001

Biomarkers	*β*	SE	Wald	HR (95% C.I.)	*P*
miR-425-5p	0.953	0.362	6.922	2.593 (1.275-5.272)	0.009
CD8 (High vs Low)	-1.127	0.522	4.663	0.324 (0.117-0.901)	0.031
CCL18 (High vs Low)	1.32	0.669	3.896	3.745 (1.009-13.893)	0.048
